# Biophysical Differentiation Between Lymphocytes from Healthy Donors, Patients with Malignant Diseases and Other Disorders

**DOI:** 10.1038/bjc.1974.81

**Published:** 1974-05

**Authors:** L. Cercek, B. Cercek, C. I. V. Franklin

## Abstract

Changes in the structuredness of the cytoplasmic matrix (SCM) of human lymphocytes induced by PHA, CaBP and EF were studied with the technique of fluorescence polarization. The study suggests that the SCM test may offer a new and fast technique for the detection of malignant growth.


Br. J. Cancer (1974) 29, 345

### BIOPHYSICAL DIFFERENTIATION BETWEEN LYMPHOCYTES FROM HEALTHY DONORS, PATIENTS WITH MALIGNANT DISEASES AND OTHER DISORDERS

L. CERCEK, B. CERCEK AND C. I. V. FRANKLIN

*From the Paterson Laboratories, Christie Hospital and Holt Radium Institute, Manchester M20 9BX*

Received 8 February 1974. Accepted 11 February 1974

**Summary.—Changes in the structuredness of the cytoplasmic matrix (SCM) of human lymphocytes induced by PHA, CaBP and EF were studied with the technique of fluorescence polarization. The study suggests that the SCM test may offer a new and fast technique for the detection of malignant growth.**

WE HAVE reported recently that normal human and chronic lymphocytic leukaemia (CLL) lymphocytes can be differentiated on the basis of changes in the structuredness of cytoplasmic matrix (SCM) induced by phytohaemagglutinin (PHA) stimulation (Cercek, Cercek and Garrett, 1974). By analogy with the effect of PHA stimulation, we expected other mitogens and/or antigens to induce changes in the SCM of lymphocytes which could be used for the differentiation between various diseases. In continuation of the previous study, we have therefore now investigated the effects of PHA and tumour antigens (Field and Caspary, 1970), *i.e.* cancer basic protein (CaBP) and encephalitogenic factor (EF), on the changes in the SCM of lymphocytes from healthy donors and patients with malignant and non-malignant disorders. The aim of this study was to find out if changes in the SCM of lymphocytes evoked by either PHA or CaBP and/or EF could be used in the detection of malignancy.

### MATERIALS AND METHODS

*Separation of lymphocytes.*—Human lymphocytes were prepared from blood collected in Searle-LH/10 lithium heparin containing vials. Ten ml samples were transferred into vials containing 0·1 g of carbonyl iron powder Type SF (GAF, Great Britain, Ltd) and rotated at 120 rev/min for 30 min. Vials were then placed into the incubator at 37°C on a magnet for 10 min. Lymphocytes in a pure state (>90%) were obtained by the Ficoll–Triosil gradient separation (Harris and Ukaejiofo, 1969). The lymphocytes were washed twice with saline and twice with TC Medium 199 (Wellcome Ltd) and resuspended in TC Medium 199 at the concentration of approximately $5 \times 10^6$ cells/ml.

*Stimulation of lymphocytes.*—Aliquots of 1 ml of lymphocyte suspensions were incubated at 37°C with either 0·1 ml of a 5 times diluted reagent grade PHA (Wellcome Ltd), 0·1 ml of CaBP solution (approximately 50 μg/ml), 0·1 ml of partly purified CaBP solution (0·5 ng/ml) or 0·1 ml of EF solution (approximately 50 μg/ml). CaBP and EF were donated to us by Dr J. P. Dickinson, MRC-Demyelinating Diseases Unit, Newcastle General Hospital.

*Measurements of SCM.*—Changes in the SCM of lymphocytes were measured with the technique of fluorescence polarization. The technique is based on the excitation of the fluorescein molecules produced by enzymatic hydrolysis of the non-fluorescing substrate, fluoresceindiacetate (FDA), in the cytoplasm with polarized light, and measurement of the degree of polarization of the emitted fluorescence. Aliquots of controls or of the incubated lymphocytes were suspended at concentrations of $3 \times 10^5$ lymphocytes/ml in 2·5 μmol/l FDA solution in phosphate buffered saline. The suspension was rapidly transferred into a 1 cm cuvette and put into the thermostated cuvette holder of the Perkin-Elmer MPF-2A fluorescence spectrophotometer fitted with the polarization accessory. Measurements were made at 27°C. Details of the experimental conditions, procedures and calculations of fluorescence polarization values, $P$, were the same as described in experiments when changes in the SCM were measured in Chinese Hamster cells (Cercek, Cercek and Ockey, 1973).

### RESULTS

The mean value of the SCM of lymphocytes from 71 healthy donors is $P = 0{\cdot}206 \pm 0{\cdot}002$ (standard error). The age and sex distribution of these donors is given in Table I. The mean value of the SCM of lymphocytes from 41 donors with malignant disease is $P = 0{\cdot}201 \pm 0{\cdot}0015$ (25 females and 16 males aged 16–85 years) and that of lymphocytes of 17 patients with non-malignant disorders is $P = 0{\cdot}188 \pm 0{\cdot}002$ (10 females and 7 males aged 18–84 years). Details of the various malignant and non-malignant cases investigated are given in Tables II and III.

Lymphocytes from healthy donors

TABLE I.—*Healthy Donors*

| Total number of donors tested with | | | | | | No. of SCM responders to | | |
|---|---|---|---|---|---|---|---|---|
| PHA | Age | Sex | CaBP | Age | Sex | PHA | CaBP | $RR_{SCM}$ |
| 71* | 18–69 | 22F 49M | 41 | 18–69 | 12F 29M | 70/71 | 1/41 | 1·28–1·60 (The one exception: 0·84) |

* Of these 41 were tested with CaBP also.

FIG. 1.—Effect of PHA on the SCM of human lymphocytes: means of 70 healthy donors (○)—and 41 donors with malignant diseases (●). Deviations indicated are standard errors of the mean.

TABLE II.—*Patients with Malignant Diseases*

| Case no. | Age | Sex | Diagnosis | Treatment before SCM test | SCM decrease induced by PHA | SCM decrease induced by CaBP | $RR_{SCM}$ |
|---|---|---|---|---|---|---|---|
| 1 | 51 | F | Carcinoma of breast | — | 0 | + | 0·70 |
| 2 | 61 | F | Carcinoma of breast, scirrhous ST2 | 250 rad | 0 | + | 0·71 |
| 3 | 63 | F | Carcinoma of breast: recurrence, metastases in bones | 1966 surgery, since 1971 hormones and chemotherapy | 0 | + | 0·75 |
| 4 | 79 | F | Transitional cell carcinoma of bladder | 500 rad | 0 | + | 0·74 |
| 5 | 60 | M | Transitional cell carcinoma of bladder, ST2 | — | 0 | + | 0·86 |
| 6 | 65 | F | Carcinoma of bladder, ST2 | — | 0 | + | 0·75 |
| 7 | 52 | F | Carcinoma of tongue | — | 0 | + | 0·71 |
| 8 | 61 | M | Adenocarcinoma of tongue, ST4 | — | 0 | + | 0·72 |
| 9 | 60 | F | Carcinoma of larynx | — | 0 | + | 0·73 |
| 10 | 51 | F | Squamous cell carcinoma of larynx, ST1 | — | 0 | + | 0·68 |
| 11 | 66 | M | Squamous cell carcinoma of larynx, ST2 | 4900 rad | 0 | + | 0·81 |
| 12 | 40 | F | Squamous cell carcinoma of cervix uteri, ST3 | — | 0 | + | 0·81 |
| 13 | 80 | F | Adenocarcinoma of cervix uteri, ST2 | — | 0 | + | 0·71 |
| 14 | 52 | F | Squamous cell carcinoma of cervix uteri, ST2 | — | 0 | + | 0·71 |
| 15 | 59 | F | Squamous cell carcinoma of cervix uteri, ST3 | — | 0 | + | 0·72 |
| 16 | 61 | F | Squamous cell carcinoma of cervix uteri, ST2B | 900 rad | 0 | + | 0·73 |
| 17 | 68 | F | Adenocarcinoma of uterus body, recurrence | Hysterectomy in 1972 | 0 | + | 0·74 |
| 18 | 30 | F | Carcinoma of ovary—late | — | 0 | + | 0·72 |
| 19 | 56 | M | Malignant melanoma (recurrence) | 430 rad | 0 | + | 0·68 |
| 20 | 79 | F | Malignant melanoma (late) | 430 rad | 0 | + | 0·71 |
| 21 | 60 | M | Squamous cell carcinoma of skin: upper lip | — | 0 | + | 0·76 |
| 22 | 72 | F | Basal cell carcinoma of skin: on temple | — | 0 | + | 0·69 |
| 23 | 70 | F | Skin intra-epidermal carcinoma, ST2 | — | 0 | + | 0·63 |
| 24 | 72 | M | Squamous cell carcinoma of upper (ST2) and lower (ST1) lip | 2000 rad to lower lip | 0 | + | 0·69 |
| 25 | 67 | M | Undifferentiated carcinoma of bronchus (early) | — | 0 | + | 0·73 |
| 26 | 67 | M | Anaplastic carcinoma of bronchus (late) | 1130 rad | 0 | + | 0·78 |
| 27 | 58 | M | Oat cell carcinoma of bronchus (early) | — | 0 | + | 0·69 |
| 28 | 44 | F | Glioblastoma of thalamus | 465 rad | 0 | + | 0·71 |
| 29 | 44 | M | Astrocytoma, Grade III | 495 rad | 0 | + | 0·82 |
| 30 | 16 | F | Osteogenic sarcoma of knee (early) | 660 rad | 0 | + | 0·75 |
| 31 | 79 | M | Osteogenic sarcoma or Paget's disease | 375 rad | 0 | + | 0·68 |
| 32 | 52 | F | Squamous cell carcinoma in neck, secondary tumour, primary tumour unknown | 500 rad | 0 | + | 0·76 |
| 33 | 68 | M | Adenocarcinoma of colon, ST2 | — | 0 | + | 0·76 |
| 34 | 66 | M | Squamous cell carcinoma of pharynx ST3 with metastases in glands | 1125 rad | Increase | + | 0·73 |
| 35 | 27 | F | Bone (Ewing's) tumour | — | 0 | + | 0·74 |
| 36 | 57 | M | Adenocarcinoma of kidney (residual disease) | Surgery | 0 | + | 0·67 |
| 37 | 74 | F | Squamous cell carcinoma of oesophagus | — | 0 | + | 0·65 |
| 38 | 66 | F | Carcinoma of thyroid with metastases | — | 0 | + | 0·80 |
| 39 | 53 | F | Reticulum cell sarcoma | — | 0 | + | 0·89 |
| 40 | 64 | M | Squamous cell carcinoma of larynx | — | 0 | + | 0·78 |
| 41 | 67 | M | Malignant melanoma | 430 rad | 0 | + | 0·90 |
| | | | *" Premalignant " conditions:* | | | | |
| | 34 | M | Polyposis coli | — | Increase | + | 0·78 |
| | 53 | M | Hyperkeratosis of skin | — | + | + | 1·08 |

TABLE III.—*Patients with Various Non-malignant Disorders*

| | | | | SCM decrease induced by | | |
|---|---|---|---|---|---|---|
| Case no. | Diagnosis | Age | Sex | PHA | CaBP | $RR_{SCM}$ |
| 1 | Multiple sclerosis | 60 | F | + | 0 | 1·30 |
| 2 | Multiple sclerosis | 53 | M | + | 0 | 1·35 |
| 3 | Infective hepatitis | 23 | F | + | 0 | 1·30 |
| 4 | Crohn's disease | 29 | F | + | 0 | 1·32 |
| 5 | Cirrhosis of liver | 51 | F | + | 0 | 1·48 |
| 6 | Cirrhosis of liver | 45 | F | + | 0 | 1·51 |
| 7 | Ulcerative colitis | 24 | M | + | 0 | 1·36 |
| 8 | Chronic bronchitis | 60 | M | + | 0 | 1·37 |
| 9 | Chronic bronchitis | 61 | M | + | 0 | 1·35 |
| 10 | Chronic bronchitis | 67 | M | + | 0 | 1·23 |
| 11 | Rheumatoid arthritis and chronic bronchitis | 57 | F | + | 0 | 1·35 |
| 12 | Rheumatoid arthritis and psoriasis | 52 | M | + | 0 | 1·29 |
| 13 | Gastric ulcer and benign enlargement of prostate | 84 | M | + | 0 | 1·36 |
| 14 | Pregnancy 35 weeks | 27 | F | + | 0 | 1·50 |
| 15 | Pregnancy 24 weeks | 20 | F | + | 0 | 1·59 |
| 16 | Pregnancy 32 weeks | 18 | F | + | 0 | 1·50 |
| 17 | Pregnancy 32 weeks | 24 | F | + | 0 | 1·57 |

FIG. 2.—Effect of CaBP on the SCM of human lymphocytes: means of 40 healthy donors (○)— and 41 douors with malignant diseases (●). Deviations indicated are standard errors of the mean.

(70/71 cases) and patients with non-malignant disorders (17/17 cases) responded to PHA stimulation with an immediate decrease in the SCM to 79% of the control value followed by a further slow decrease with time of incubation (Fig. 1). In contrast, lymphocytes from all donors with malignant diseases (Tables II and IV) did not respond to PHA stimulation with a decrease in the SCM for up to 180 min of incubation (Fig. 1).

Lymphocytes from healthy donors (40/41 cases) and from all patients with non-malignant disorders did not respond

TABLE IV.—*SCM Response of Lymphocytes from Different Healthy Donors and Patients with Malignant and Non-malignant Disorders to Myelin Protein (EF)*

| Case no. | Diagnosis | Age | Sex | SCM decrease induced by PHA | SCM decrease induced by EF | $RR_{SCM}$ ($P_{EF}/P_{PHA}$) |
|---|---|---|---|---|---|---|
| 1 | Cancer of breast | 51 | F | 0 | + | 0·70 |
| 2 | Cancer of breast | 61 | F | 0 | + | 0·71 |
| 3 | Cancer of bladder | 79 | F | 0 | + | 0·73 |
| 4 | Cancer of tongue | 52 | F | 0 | + | 0·71 |
| 5 | Cancer of larynx | 60 | F | 0 | + | 0·70 |
| 6 | S.C.C. skin | 60 | M | 0 | + | 0·76 |
| 7 | Healthy donor | 37 | M | + | 0 | 1·44 |
| 8 | Healthy donor | 21 | M | + | 0 | 1·53 |
| 9 | Healthy donor | 20 | M | + | 0 | 1·47 |
| 10 | Healthy donor | 26 | F | + | 0 | 1·42 |
| 11 | Healthy donor | 43 | F | + | 0 | 1·47 |
| 12 | Healthy donor | 43 | M | + | 0 | 1·42 |
| 13 | Multiple sclerosis | 60 | F | + | + | 1·00 |
| 14 | Multiple sclerosis | 53 | M | + | + | 0·99 |

FIG. 3.—Effect of EF on the SCM of human lymphocytes: ○—6 healthy donors; ●—6 donors with malignant disease.

to CaBP stimulation for up to 180 min of incubation (Fig. 2 and Tables I and III). Similarly, the lymphocytes from healthy donors (6/6 cases) did not respond to EF stimulation (Fig. 3, Table IV). However, lymphocytes from 2 donors with a demyelinating disease (multiple sclerosis) responded to EF stimulation with a decrease in the SCM (Table IV).

Lymphocytes from donors with malignant diseases (41/41 cases) responded to CaBP and to EF (6/6 cases) stimulation with an immediate decrease in the SCM to 76% of the control value followed by a further slow decrease with time of incubation (Fig. 2, 3). In 2 cases of premalignant conditions, *i.e.* polyposis coli (familial cancer) and hyperkeratosis of the skin, the lymphocytes responded to CaBP stimulation with a decrease in the SCM to 91% of the control value.

To express the response of the lymphocytes to CaBP and PHA stimulation as a single parameter we have calculated the "SCM Response Ratio" ($RR_{SCM}$). The $RR_{SCM}$ value is the ratio of the degree of fluorescence polarization obtained after CaBP stimulation, $P_{CaBP}$, over that after PHA stimulation, $P_{PHA}$, both measured at comparable times after 30 to 100 min of incubation:

$$RR_{SCM} = P_{CaBP}/P_{PHA}$$

A histogram of $RR_{SCM}$ values for lymphocytes from healthy donors, donors with malignant diseases and non-malignant disorders is shown in Fig. 4. It can be seen that the lymphocytes from patients with malignant diseases can be differentiated easily from the lymphocytes of other donors on the basis of the $RR_{SCM}$ parameter.

### DISCUSSION

The physical phenomenon of changes in the SCM appears to be a sensitive monitor of the ability of lymphocytes to respond to mitogenic and/or antigenic stimuli (Cercek *et al.*, 1974). The summary of the results in Fig. 1, 2 and 3 shows that lymphocytes from donors with various malignant diseases (Table II) can be differentiated from lymphocytes of healthy donors and from donors with various non-malignant disorders (Table III) which in other tests for the diagnosis of human cancer gave false positive results (Lawrence and Neville, 1972; Tee, 1973; Field, Caspary and Smith, 1973). The lymphocytes from cancer patients respond to stimulation by CaBP (or EF) but not to that by PHA, whereas the lymphocytes from healthy donors and patients with non-malignant disorders respond to stimulation by PHA but not to that by CaBP (or EF, except 2 cases of multiple sclerosis).

A clear-cut separation of the malignant and non-malignant cases is also obtained by the frequency distribution of the $RR_{SCM}$ values (Fig. 4), which measure the magnitude of the response both to PHA and CaBP stimulation and thus could become diagnostically useful. In the progression from the normal to "premalignant" to malignant state the $RR_{SCM}$ value appears to decrease (progressively?) from values greater than 1 to smaller than 1. For example, a case of hyperkeratosis of the skin whose lymphocytes responded to PHA and CaBP stimulation with a 16% and 9% decrease in the SCM, respectively, gave a low $RR_{SCM}$ value of 1·08, but fully developed cancers of the skin gave $RR_{SCM}$ values from 0·63 to 0·69 (Table II).

Similarly, in a case of the familial condition, polyposis coli, the lymphocytes responded to CaBP stimulation with a small (9%) decrease in the SCM; however, on stimulation with PHA the response was a 17% *increase*, similar to that observed in CLL lymphocytes (Cercek *et al.*, 1974). The $RR_{SCM}$ value in this case was 0·78.

Unlike with the macrophage migration inhibition test (Field *et al.*, 1973), the lymphocytes from 2 cases of multiple sclerosis responded to PHA and CaBP in the same way as those from healthy donors (Table III) but responded to EF

FIG. 4.—Frequency distribution of the SCM Response Ratio ($RR_{SCM}$) of lymphocytes from healthy donors, patients with malignant diseases and various other disorders: [vertical hatching]—malignant diseases; [stippled]—healthy donors; 1. multiple sclerosis; 2. infective hepatitis; 3. Crohn's disease; 4. cirrhosis of liver; 5. pregnancy (24–35 weeks); 6. ulcerative colitis; 7. chronic bronchitis; 8. rheumatoid arthritis combined with chronic bronchitis and psoriasis; 9. peptic ulcer and benign enlargement of prostate; 10. hyperkeratosis of the skin; 11. polyposis coli; 12. normal donor (the exception).

with a decrease in the SCM similar to that observed in cancer patients. This observation suggests the possible usefulness of the SCM test in differentiating demyelinating conditions from malignancies of the central nervous system.

Among the 71 healthy donors we found only one (a 59-year old male) whose lymphocytes responded to PHA and CaBP stimulation in the same way as lymphocytes from donors with cancer. The $RR_{SCM}$ value was 0·91 and 8 weeks later it was 0·84. Clinical examination did not reveal any obvious sign of malignant process. Further studies on the correlation between the values of $RR_{SCM}$ and early stages of malignant growth should help to decide if such exceptions are "false positives" or cases of early malignant growth which elude the macroscopic methods of the routine clinical examinations.

From the magnitude, mode and time dependence of the response it appears that the same type and size of cell population (? T cell) is involved in the change of SCM induced by PHA and CaBP. Furthermore, EF may carry 2 types of determinants, one recognized by the receptors of lymphocytes from patients with cancer, the other by lymphocytes from patients with multiple sclerosis.

The present study suggests that the SCM test may offer a new and fast technique for the detection of a malignant growth. A long-term study on a larger number of cases is needed to explore the applicability of the SCM test and of the $RR_{SCM}$ parameter for diagnostic and prognostic purposes.

We are grateful to Professor L. G. Lajtha for his encouragement and suggestions during the progress of this study and in the preparation of the manuscript. We thank Dr J. P. Dickinson for the donation of the CaBP and EF, Professor E. C. Easson and the consultants of the Christie Hospital and of the Withington Hospital, Manchester, for blood samples and access to patients, and Miss K. Pimblett for her valuable technical assistance in the preparation of lymphocytes.

This work was supported by grants from the Cancer Research Campaign and the Medical Research Council.

### REFERENCES

CERCEK, L., CERCEK, B. & GARRETT, J. V. (1974) Biophysical Differentiation between Normal Human and Chronic Lymphocytic Leukaemia Lymphocytes. In *Lymphocyte Recognition and Effector Mechanisms* Ed. K. Lindahl-Kiessling and K. Osoba. New York, London: Academic Press. p. 553.

CERCEK, L., CERCEK, B. & OCKEY, C. H. (1973) Structuredness of the Cytoplasmic Matrix and Michaelis-Menten Constants for the Hydrolysis of FDA during the cell cycle in Chinese Hamster Ovary Cells. *Biophysik*, **10**, 187.

FIELD, E. J. & CASPARY, E. A. (1970) Lymphocyte Sensitisation: an *in vitro* Test for Cancer. *Lancet*, ii, 1337.

FIELD, E. J., CASPARY, E. A. & SMITH, K. S. (1973) Macrophage Electrophoretic Mobility (MEM) Test in Cancer: A critical Evaluation. In *Immunology of Malignancy*. Eds. M. Moore, N. W. Nesbitt and Mary V. Haigh. *Br. J. Cancer*, **28**, Suppl. I, 208.

HARRIS, R. & UKAEJIOFO, E. O. (1969) Rapid Preparation of Lymphocytes for Tissue-Typing. *Lancet*, ii, 327.

LAWRENCE, D. J. R. & NEVILLE, A. M. (1972) Foetal Antigens and their Role in the Diagnosis and Clinical Management of Human Neoplasms: A Review. *Br. J. Cancer*, **26**, 335.

TEE, D. E. H. (1973) Clinical Evaluation of the Makari Tumour Skin Test. In *Immunology of Malignancy*. Ed. M. Moore, N. W. Nesbitt and Mary V. Haigh. *Br. J. Cancer*, **28**, Suppl. 1, 187.


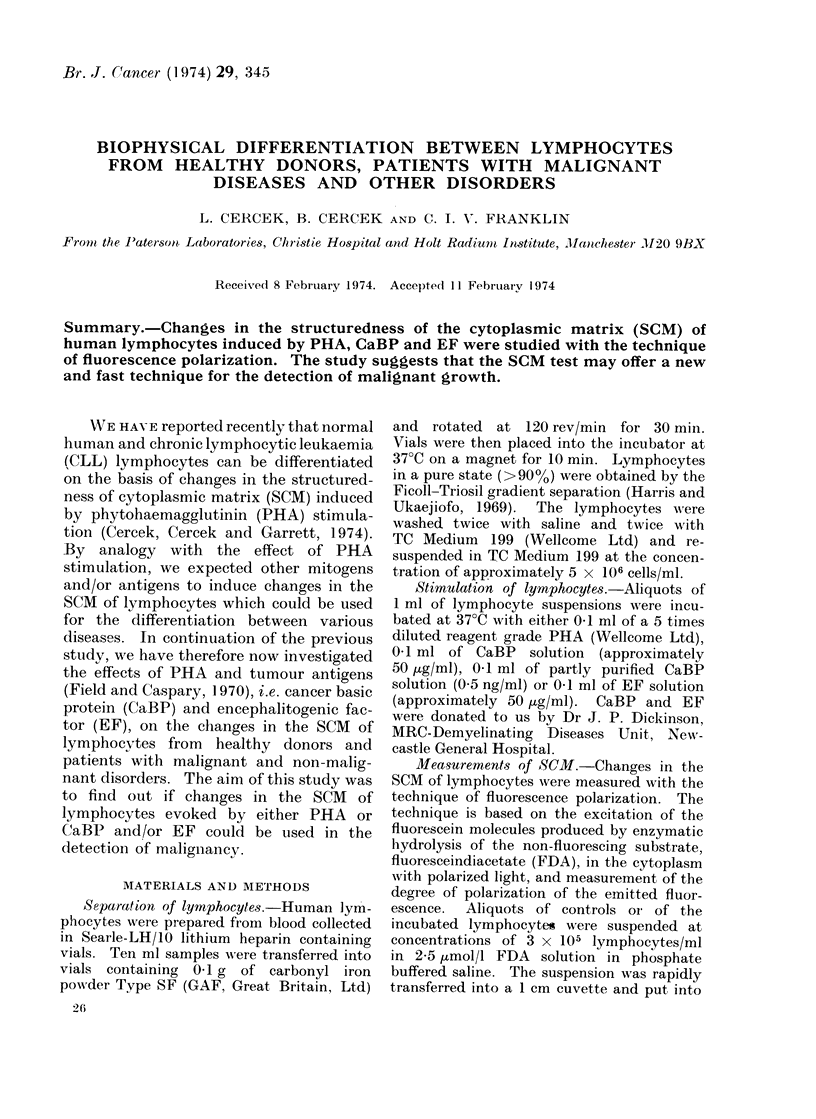

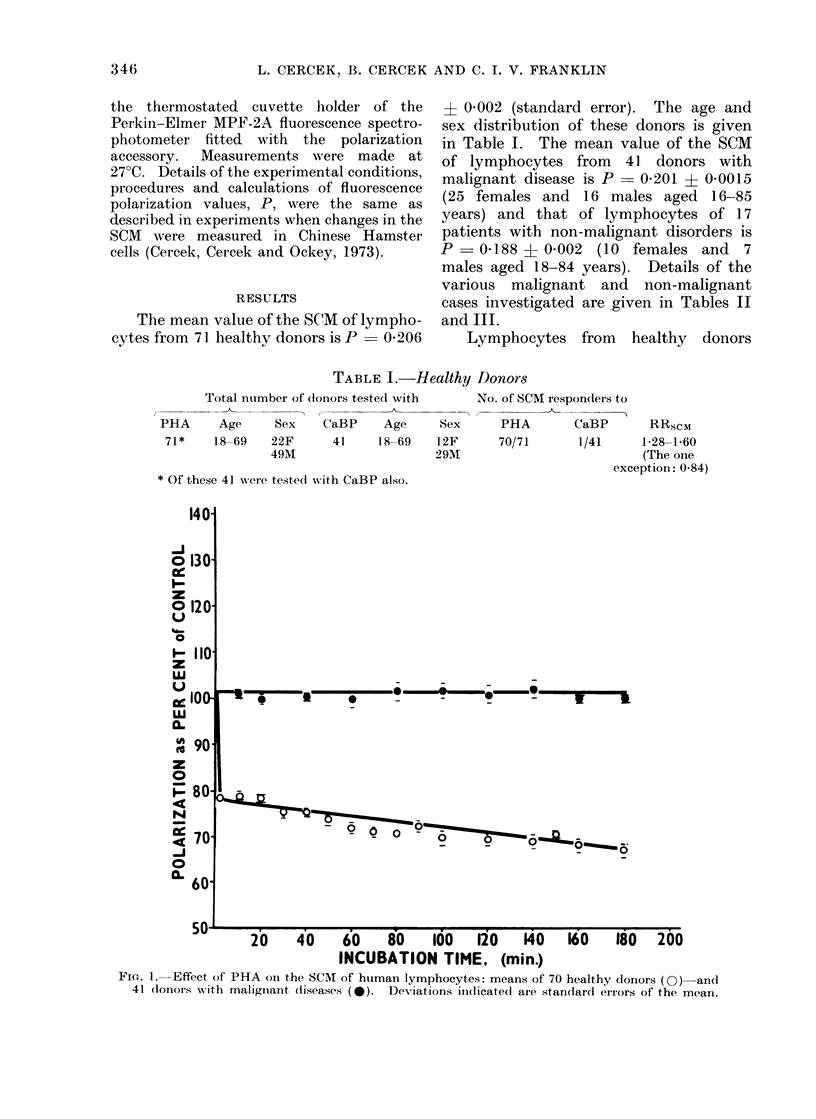

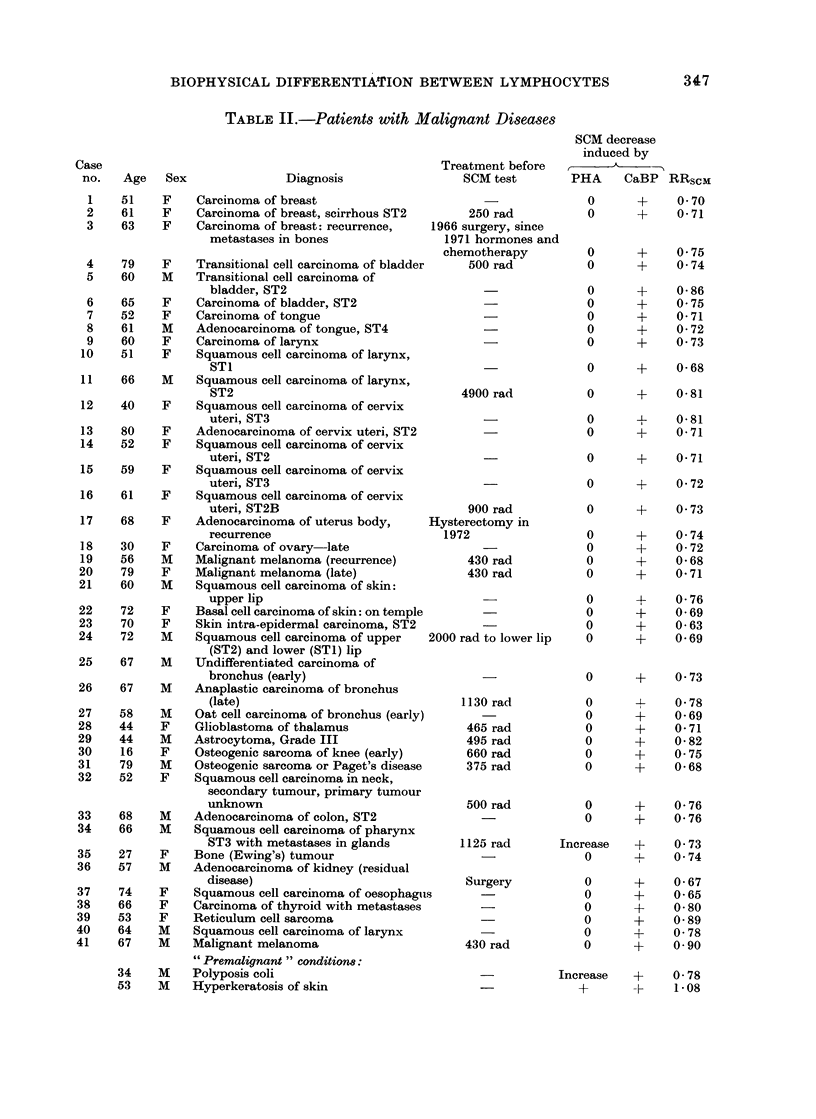

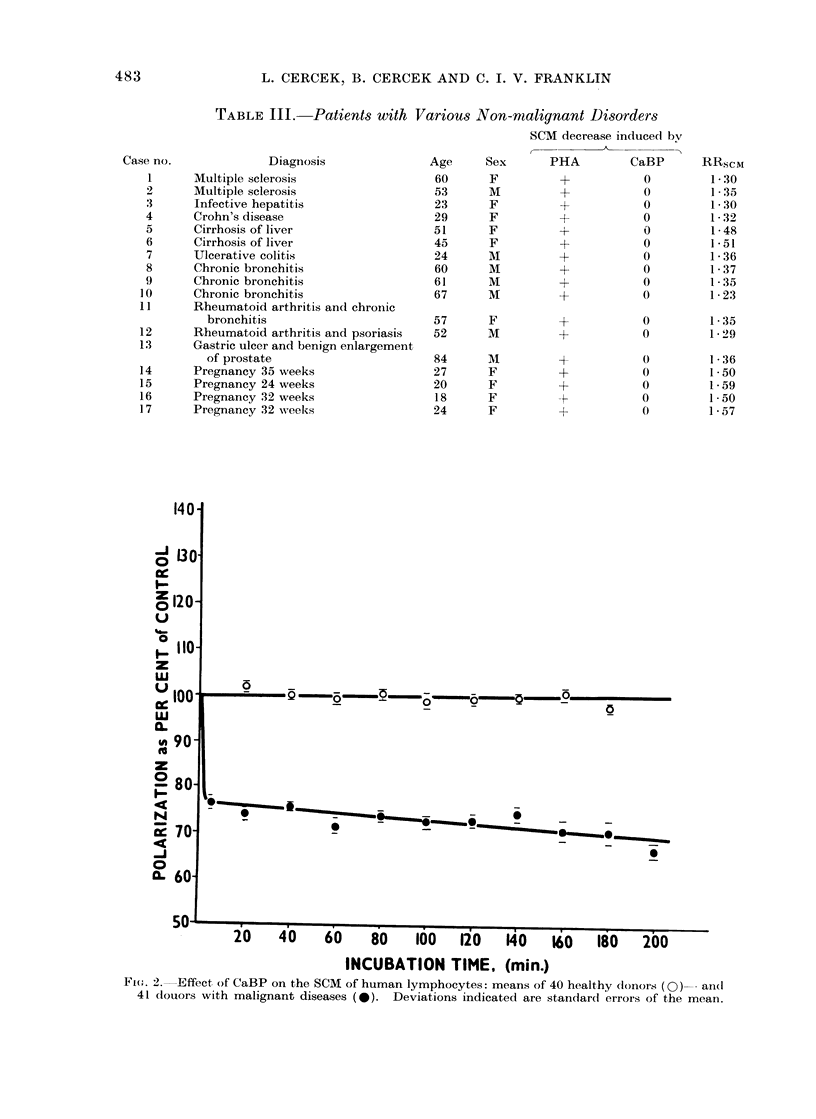

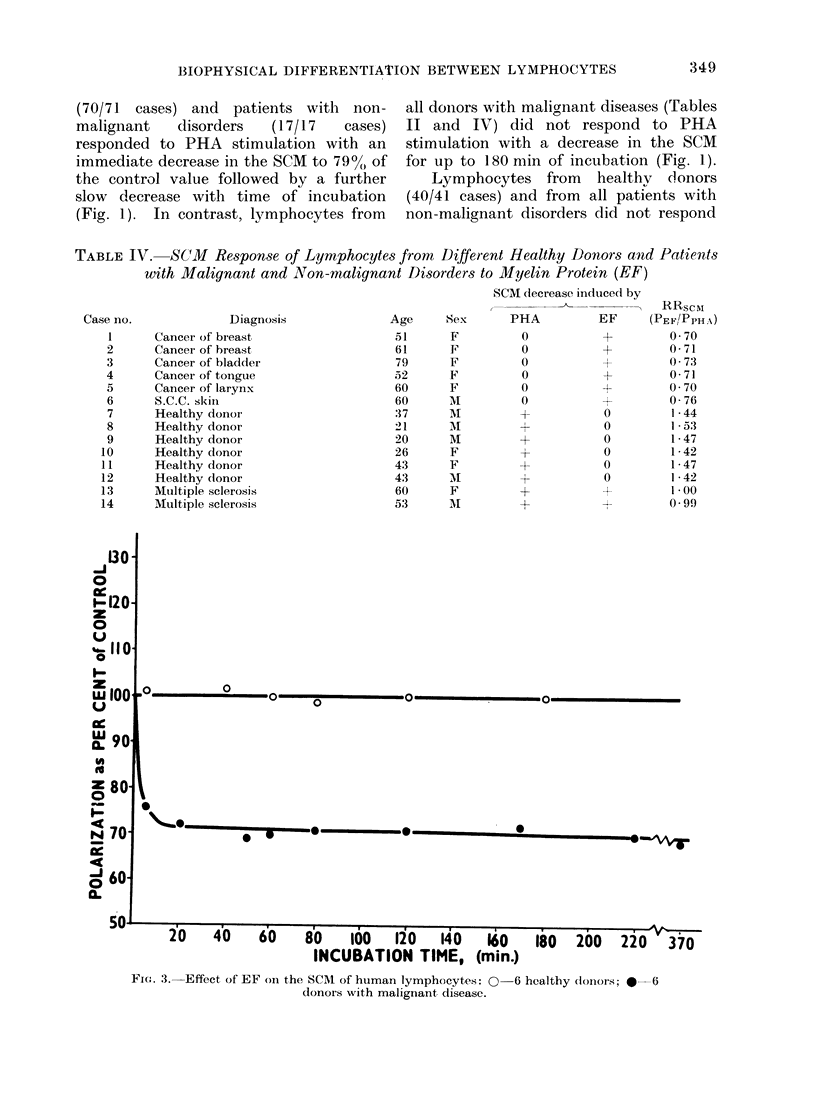

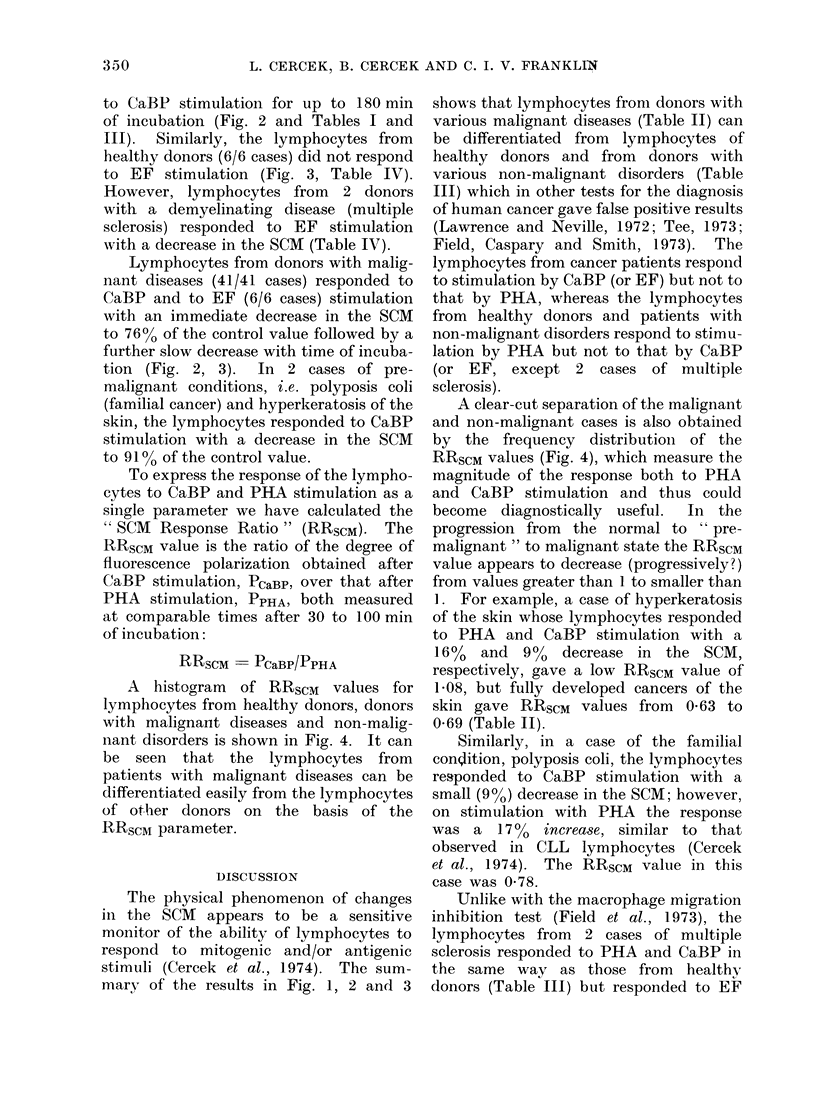

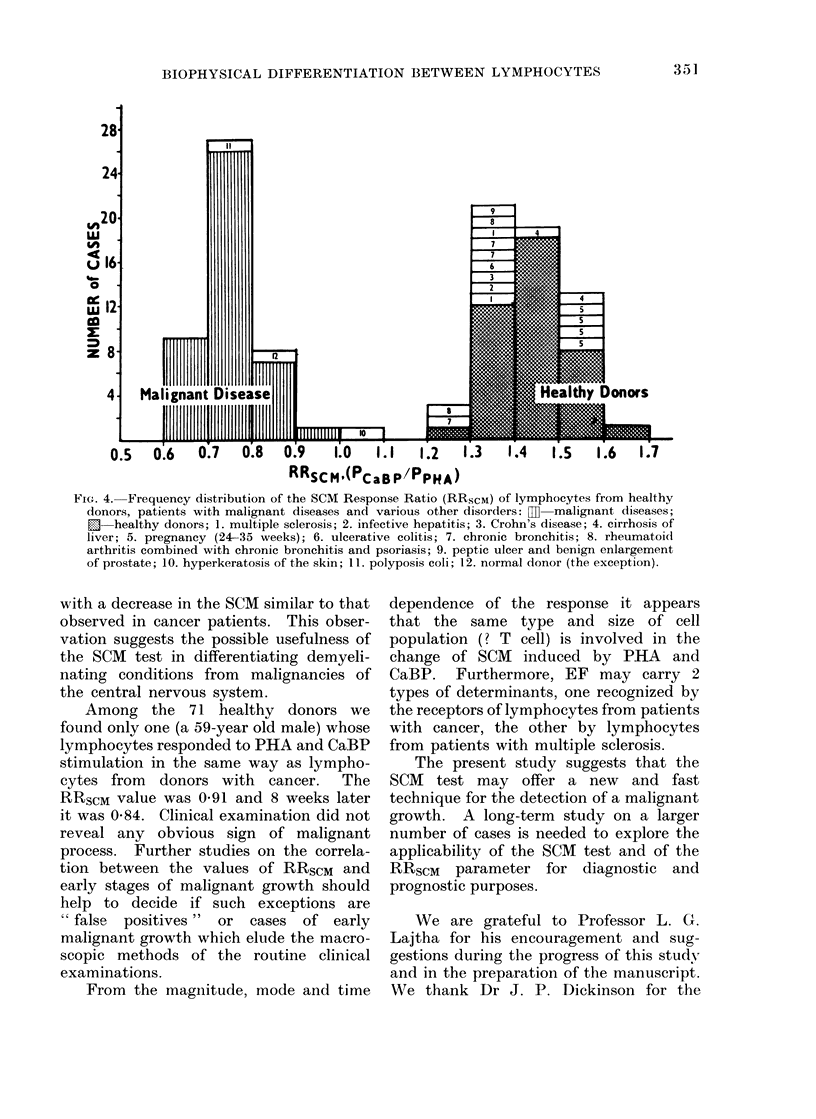

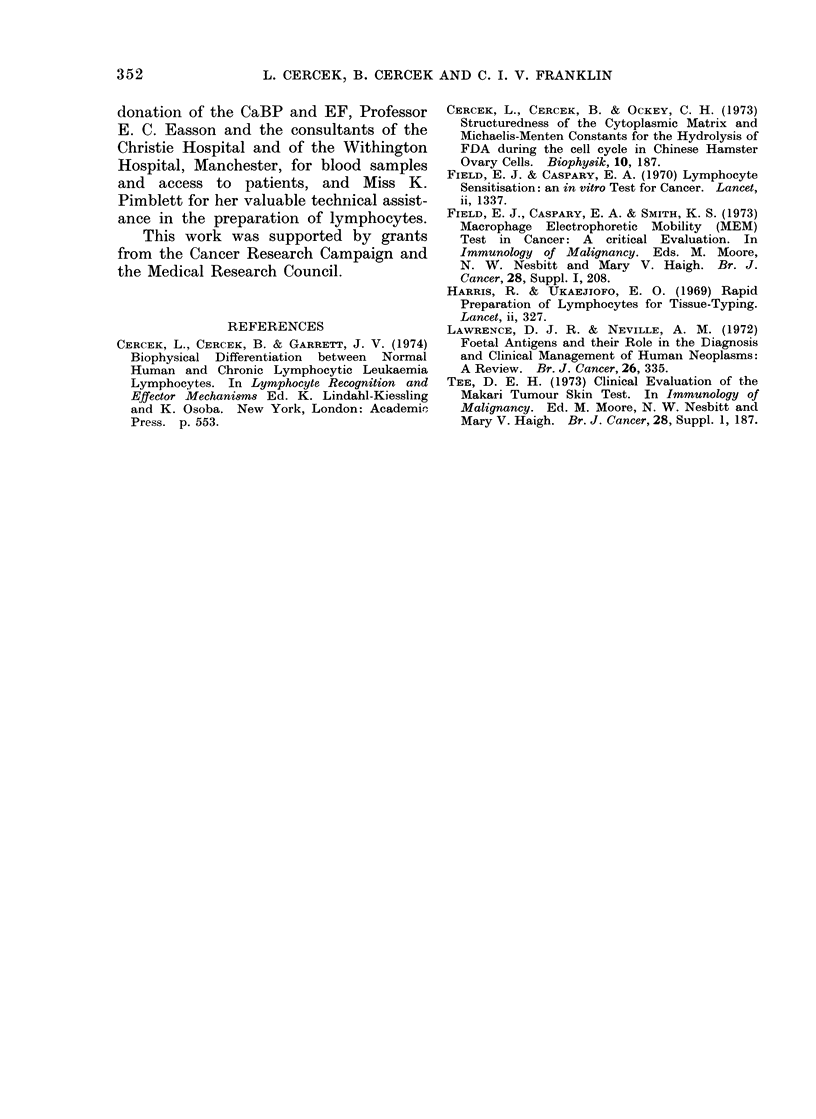


## References

[OCR_00964] Cercek L., Cercek B., Ockey C. H. (1973). Structuredness of the cytoplasmic matrix and Michaelis-Menten constants for the hydrolysis of FDA during the cell cycle in Chinese hamster ovary cells.. Biophysik.

[OCR_00971] Field E. J., Caspary E. A. (1970). Lymphocyte sensitisation: an in-vitro test for cancer?. Lancet.

[OCR_00984] Harris R., Ukaejiofo E. O. (1969). Rapid preparation of lymphocytes for tissue-typing.. Lancet.

[OCR_00989] Laurence D. J., Neville A. M. (1972). Foetal antigens and their role in the diagnosis and clinical management of human neoplasms: a review.. Br J Cancer.

[OCR_00995] Tee D. E. (1973). Clinical evaluation of the Makari tumour skin test.. Br J Cancer Suppl.

